# Marsupialization of a Large Dentigerous Cyst in the Mandible: A Case Report

**DOI:** 10.7759/cureus.27340

**Published:** 2022-07-27

**Authors:** Ali Mahfuri, Khaldoun Darwich, Ahmad Al Manadili

**Affiliations:** 1 Oral and Maxillofacial Surgery, Damascus University, Faculty of Dental Medicine, Damascus, SYR; 2 Oral and Maxillofacial Pathology, Damascus University, Faculty of Dental Medicine, Damascus, SYR; 3 Oral and Maxillofacial Pathology, International University for Science and Technology, Daraa, SYR

**Keywords:** cone-beam conventional tomography (cbct), orthopantomogram (opg), mandible, impacted tooth, marsupialization, dentigerous cyst (dc)

## Abstract

Dentigerous cysts (DCs) are one of the most common cysts in the oral and maxillofacial region, and they are often discovered by chance in young people. The methods of treatment differ according to the size they reach, but the prognosis and results of the treatment are generally good. DCs are often associated with impacted teeth, especially mandibular third molars and maxillary canines, and they are usually discovered when they reach large sizes or get infected after they have caused great absorption and destruction of the surrounding cortical bone and displacement in adjacent teeth.

This case report expresses the importance of conservative treatment of large oral cysts (by marsupialization) in the preservation of jaw bones, in a young female child of 12 years, with a DC associated with an impacted second permanent lower molar. This cyst occupied nearly half of the mandible with the danger of causing more harm to the jaw. In brief, marsupialization is a very effective method of treatment for DCs, especially those that reach very large volumes.

## Introduction

A dentigerous cyst is an extension of the dental follicle [1], more than 3-4 mm in width [2-3], which surrounds the crown of the impacted tooth during its eruption and attaches to its neck. It has a male predilection between the ages of 20 and 50, and its peak incidence is in the third decade. According to the location of radiolucency around the crown of an unerupted tooth, there are three main types of dentigerous cysts: central, lateral, and circumferential type [1,3].

When DCs reach large volumes in size, they cause eggshell crackling on clinical examination, palpation, and root resorption, but neurosensory deficits are not common [4]. Frequently, it is discovered by chance during routine radiographic imaging. If it is not extended to large volumes, it causes pathologic fracture to the jaw bone or gets infected. Possible complications of dentigerous cysts include bone deformation, fracture and destruction, permanent teeth loss, and development of ameloblastoma [1-3].

Dentigerous cysts appear radiographically as a radiolucent lesion, round or ovoid, with well-defined borders around the crowns of unerupted teeth [5]. The differential diagnosis for dentigerous cysts on plain radiograph includes odontogenic keratocyst (OKC), unicystic ameloblastoma (UA), and ameloblastic fibroma [6].

The principles of treatment of dentigerous cysts are enucleation (excision), marsupialization [1,7], and decompression [8]. Enucleation (excision) is indicated when there is no danger of damaging anatomical structures like the inferior alveolar nerve [9]. Marsupialization is a very beneficial method for treating oral cysts because it preserves the natural structures and is a minimally invasive surgery [10] but with different treatment methods. It has a good warning and does not relapse [1].

Marsupialization means the conversion of the cyst into a pouch [11] by making a window in the wall of the cyst, linking the inner epithelial lining with oral mucosa to reduce fluid pressure in the cyst on its bony walls, and permitting the oral mucosa to extend and grow into the cystic lumen. This formed cavity is filled with gauze [2,12].

Consequently, the jaw bone starts to remodel over a long period of time, causing a reduction in cystic volume to the extent of permitting it to enucleate or to leave it to heal completely [2,12].

This article was previously presented as an abstract on the Authorea website (10.22541/au.165494343.36193429/v10).

## Case presentation

A female child, 12 years of age, came to the oral and maxillofacial surgery department in the faculty of dentistry at Damascus University with an enlargement on the left side of the face.

**Figure 1:**
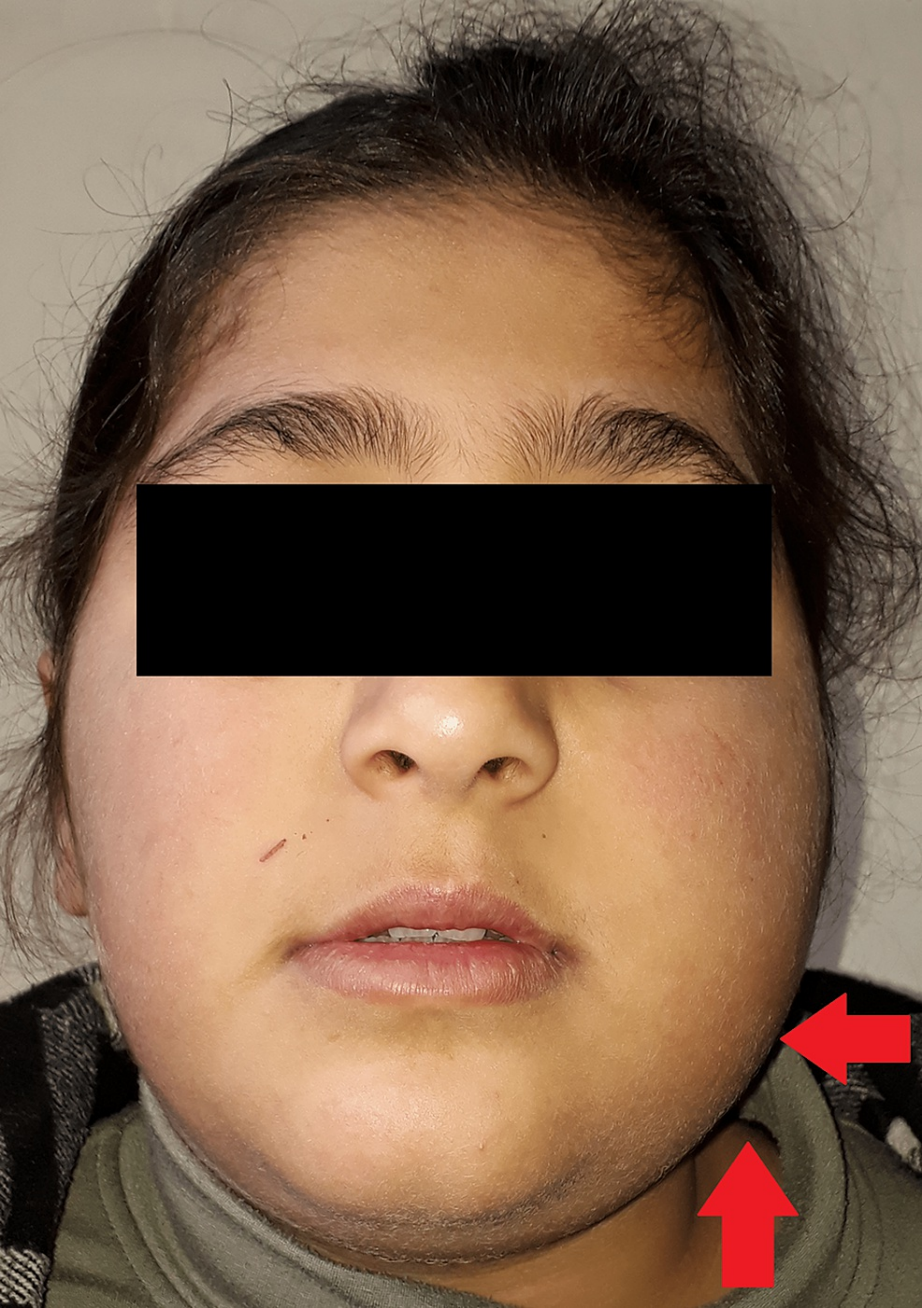
Extraoral appearance

The clinical examination is conducted for the patient and appears a tough enlargement on the left side of the face, extending from the body of the mandible to almost the ramus with limitations in mouth opening (Figure 1).

The intraoral examination showed the presence of a large tough enlargement in the lower left buccal vestibule, the absence of a lower left permanent second molar with obvious movement in the lower left permanent first molar, and the premolars with the absence of carious lesions on teeth or any inflammatory appearance on the covered gingiva.

On aspiration through the cyst wall, a clear yellow fluid is visible.

**Figure 2:**
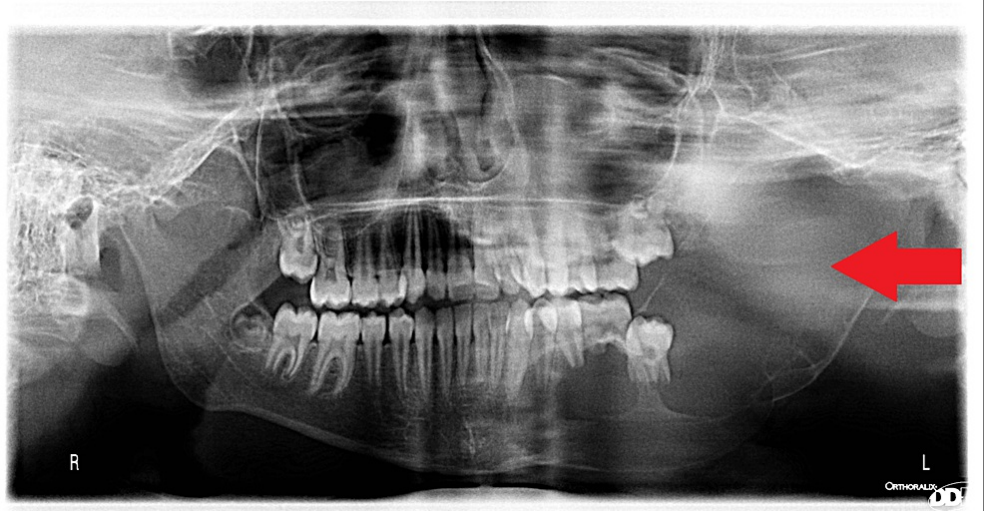
OPG shows the extension of the lesion in the mandible Radiolucent lesion occupying nearly half the volume of the mandible on the left side, showing clear great bone absorption in the mandible. OPG: orthopantomogram

Orthopantomogram (OPG) and cone beam conventional tomography (CBCT) show the presence of a large radiolucent cystic lesion with radiopaque borders involving a large volume of the mandible and extending from the lower molar to include the entire volume of the mandible and extending from the lower left canine to take the entire volume of the body of the mandible and ramus toward the coronoid and condylar processes, with radiopaque borders and an impacted second molar in the cyst (Figure 2).

**Figure 3:**
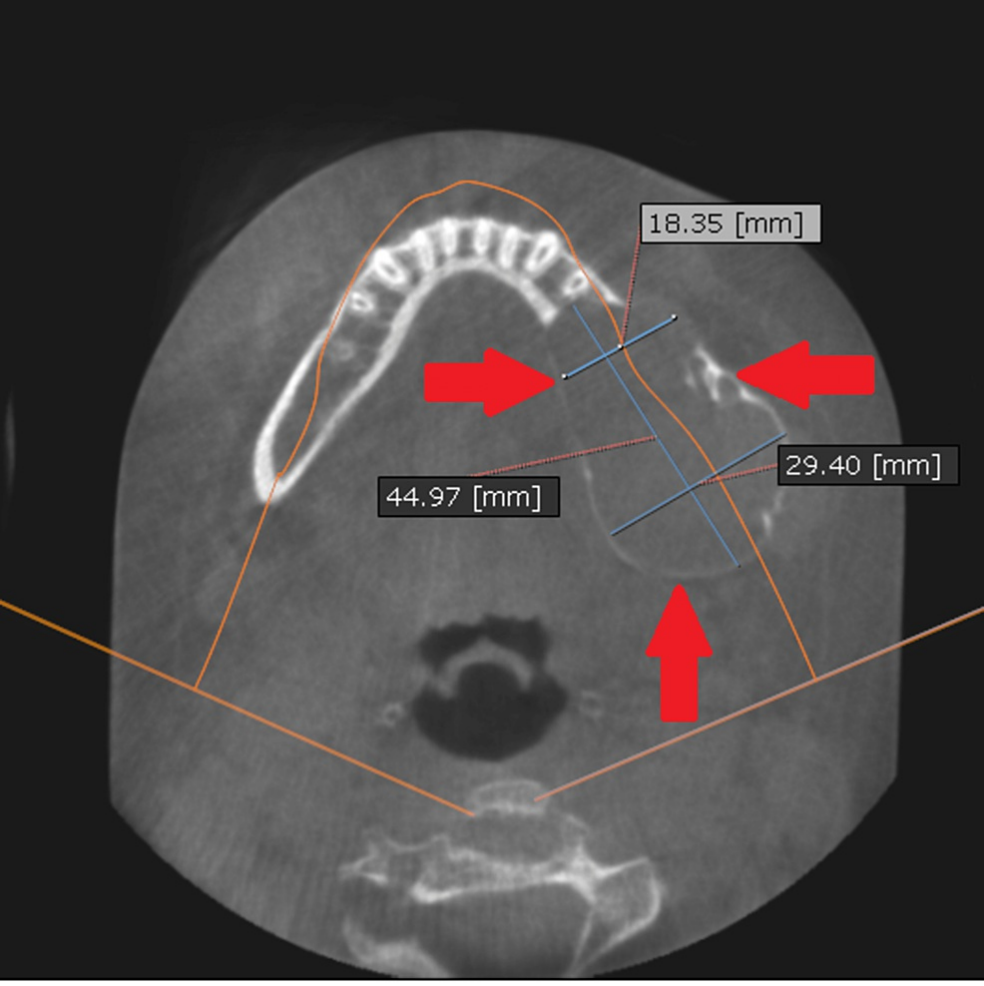
Axial section of the affected mandible Significant expansion and absorption in the buccal and lingual plates of the mandible are clearly noted.

**Figure 4:**
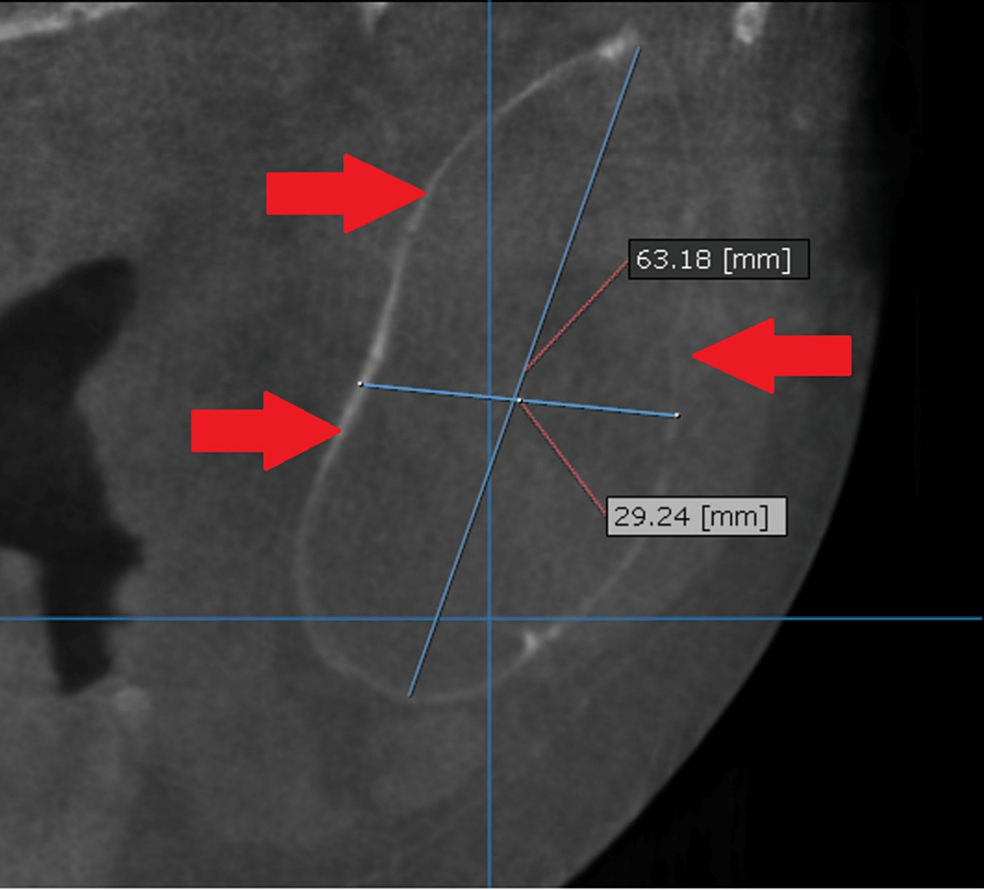
Coronal section of the affected mandible shows the huge extension and thinning of the mandible Significant expansion and absorption in the buccal and lingual plates of the mandible are clearly noted.

Sections of CBCT confirmed the extreme thinning and extension of the buccal and lingual plates of the mandible from the left side (Figures 3-4).

After studying the case, it was decided to do the surgery under general anesthesia, and the selected treatment was marsupialization.

Surgical steps

**Figure 5:**
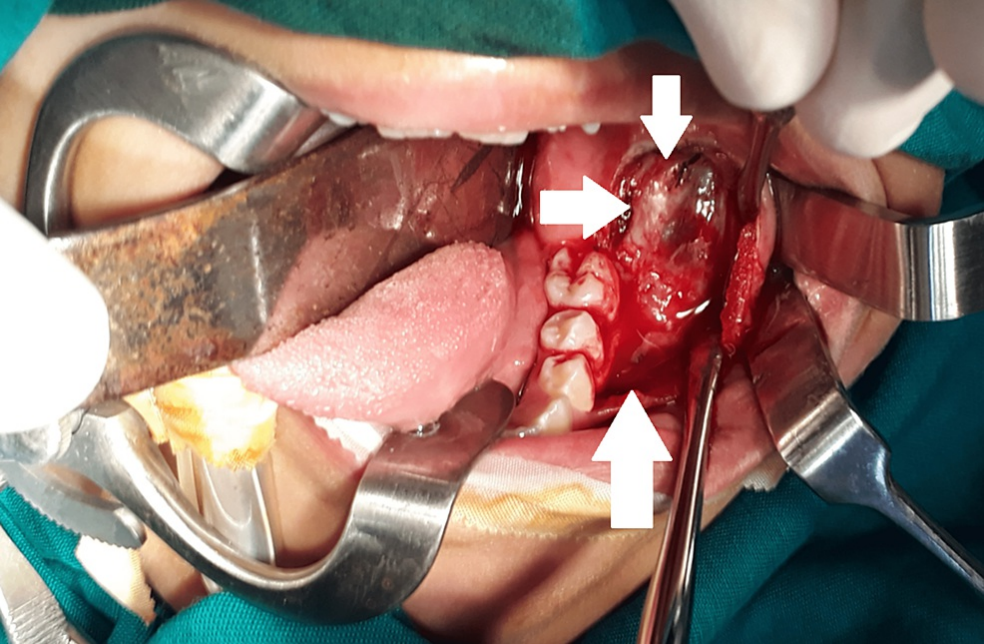
The surgical field after the elevation of the full mucoperiosteal flap Clinically, severe thinning of the buccal cortical bony plate is clearly observed.

**Figure 6:**
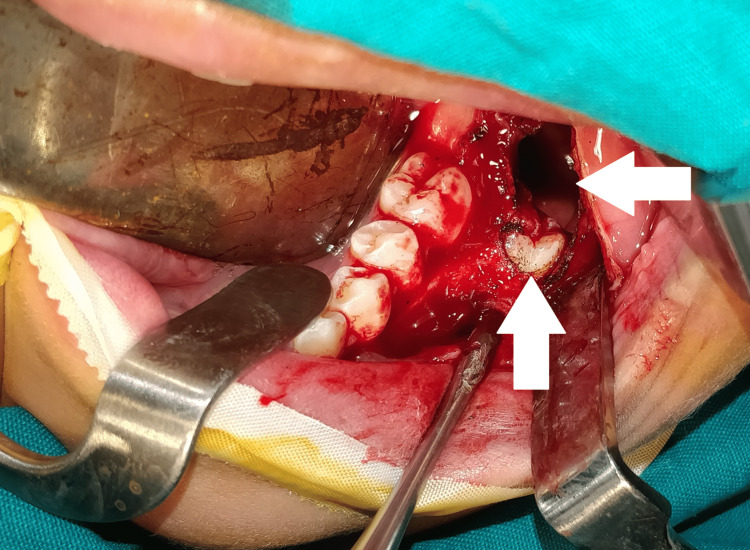
The separation of the bony window and exposure of the impacted molar Creating a wide opening for the cyst for the easy placement of the iodine gauze and preventing it from closing after a short period

**Figure 7:**
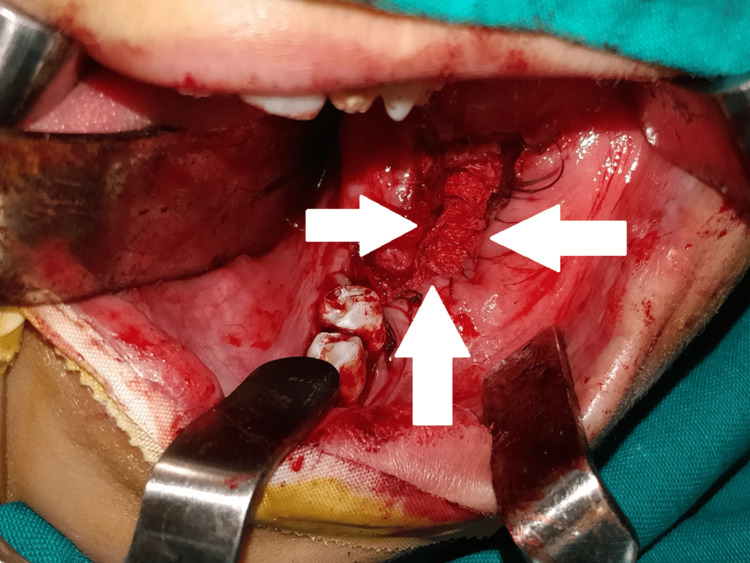
Suturing of the cystic epithelium with oral mucosa Extraction of the impacted second and the erupted first molar, filling the cyst with iodine gauze, and suturing the cystic epithelium with oral mucosa to convert the cyst to a pouch

The patient was prepared for the surgery after conducting all necessary consultations. First, exposure and disinfection of the surgical field clarified the disappearance of the second permanent molar. Then, the elevation of the full mucoperiosteal flap showed the extreme thinning of the buccal mandibular plate (Figure 5). After that, the bony window was separated from the cystic wall and the impacted lower molar was exposed (Figure 6). Then, the impacted lower molar and lower first permanent molar were extracted, the cystic lumen was filled with iodine gauze, and the oral mucosa and cystic epithelium were sutured with PLG (poly-DL-lactide/glycolide) absorbable suture (Figure 7).

**Figure 8:**
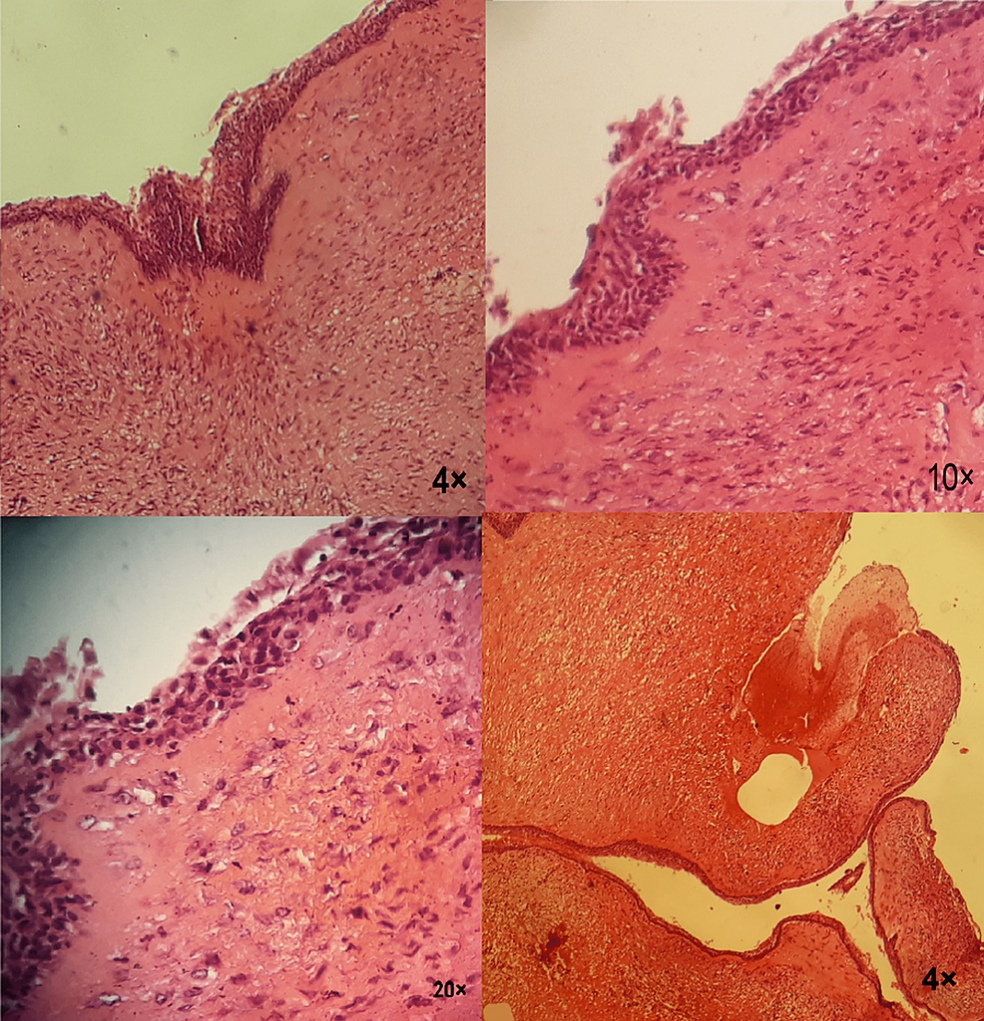
Microscopic view: pathological study confirms that the lesion is a dentigerous cyst The wall of the dentigerous cyst is lined by thin epithelial layers of undifferentiated cells, and the fibrous cyst wall is relatively inflamed. This is evident in parts a, b, and c of the image at different magnifications. Part d of the image shows a large part of the cyst wall.

A surgical biopsy was done and the result of the pathological examination confirmed the diagnosis (dentigerous cyst). The microscopic view shows the wall of the dentigerous cyst lined by thin epithelial layers of undifferentiated cells. The fibrous cyst wall was relatively inflamed (Figure 8).

**Figure 9:**
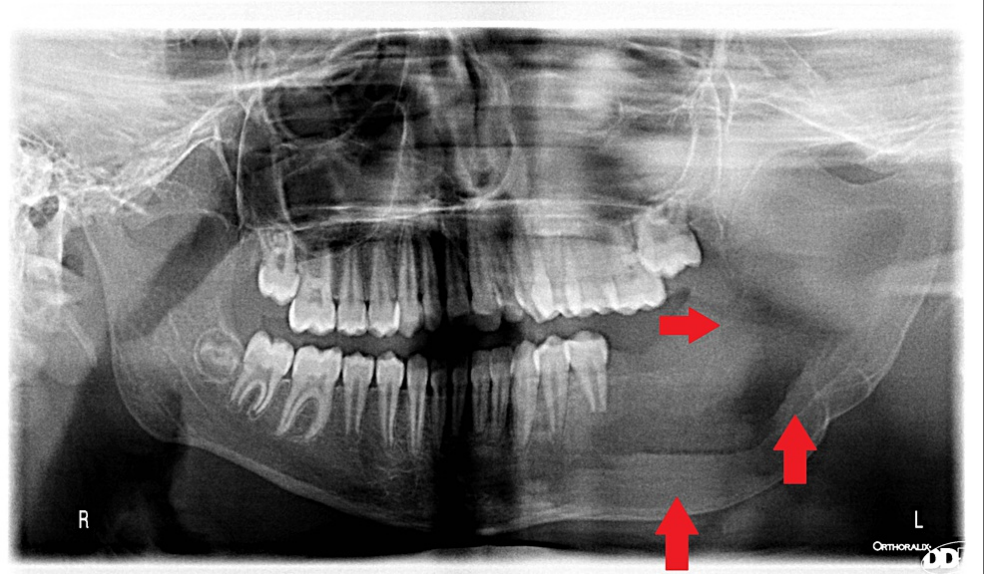
Panoramic view one week postoperatively The reason for the rapid bone restoration may be attributed to the young age of the patient.

An OPG after one week of surgery shows rapid formation of radiopacity and decrease in gauze volume during gauze replacement clinically (Figure 9).

**Figure 10:**
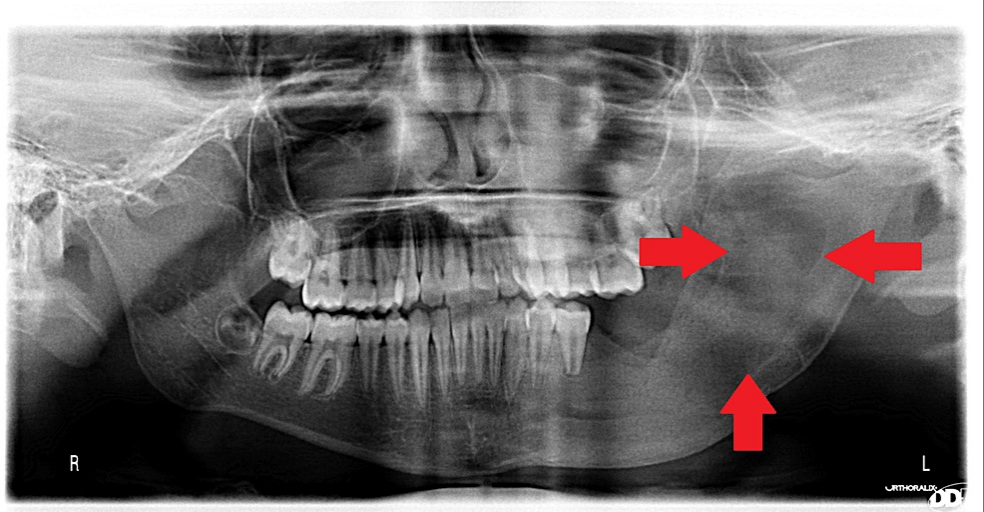
1 month postoperatively. It is noticed that within one month the size of the cyst has reduced by half.

**Figure 11:**
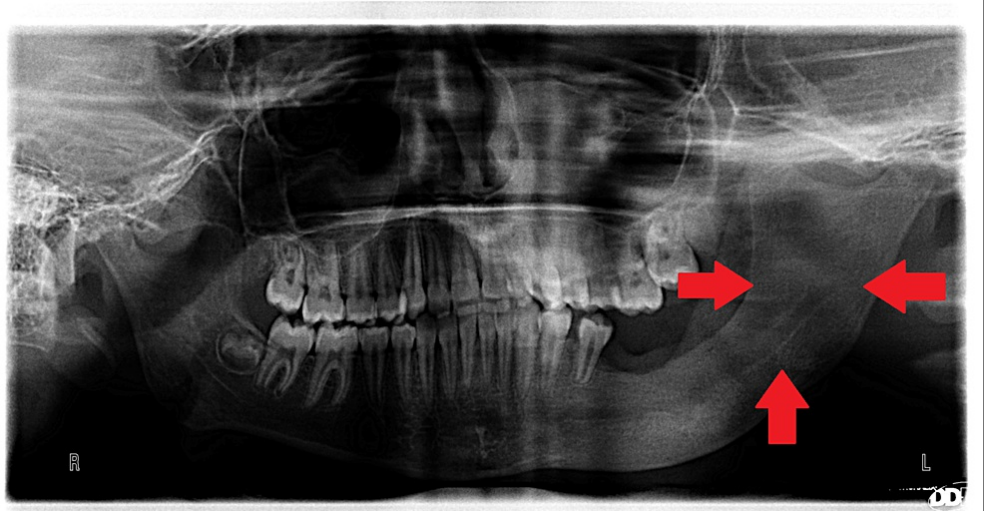
Three months postoperatively Notice after three months the approximate demise of the cyst.

**Figure 12:**
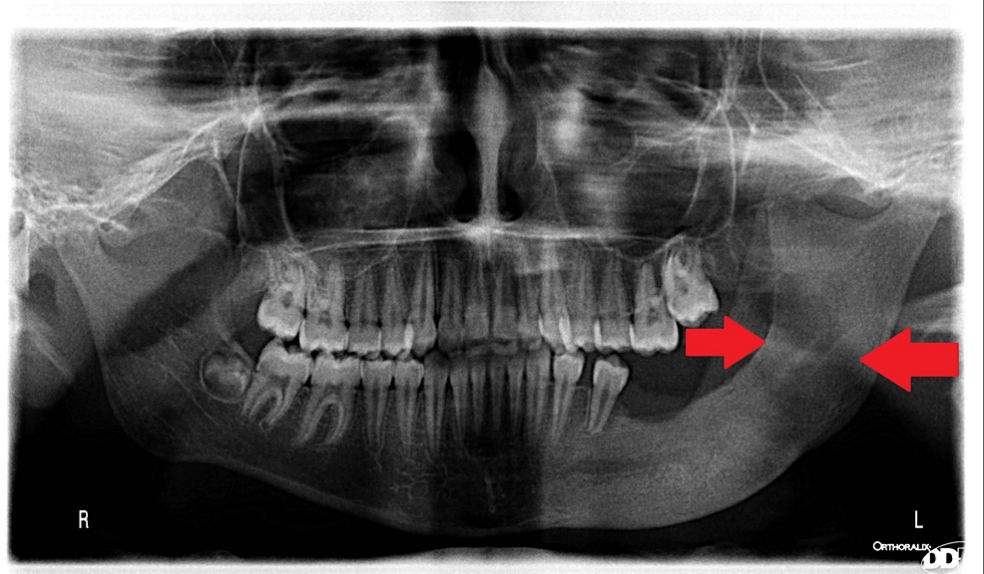
Six months postoperatively After six months, complete recovery and remodeling of the mandible are noted.

Follow-up of the case showed rapid reduction in cystic volume and formation of bone in a very short period of observation. Figures 10-12 show panoramic photos one week, one month, three months, and six months after surgery, respectively.

## Discussion

This case is characterized by the fact that the cyst reaches a large size that nearly occupies half of the mandible and causes extreme thinning in both buccal and lingual bony plates, and in rare cases, lesions like this are neglected to reach that volume. So marsupialization helped in preserving the safety of the mandible and inferior alveolar neurovascular bundle and saved the child from a more aggressive surgery to treat the lesion. Some practitioners advocate the use of the decompression method, which means making a small window in the cystic wall (similarly to marsupialization) and then using a tube (or stent) to enter the cyst through that hole [13]. While others use cystectomy (enucleation) of the lesion, which is more aggressive and takes different risks than in our case, like inferior alveolar nerve and jaw fracture [14].

So, because of the large size of the cyst in this case and because it is containing important structures, such as the inferior alveolar nerve and its vascular bundle and the proximity of the cystic borders to the condyle, the most appropriate treatment was marsupialization. Despite the long period of observation and treatment, the result is considered perfect according to the large extension of the cyst, which may be attributed to the skill of the surgical team, continuous weekly follow-up for the patient, and the high capacity for remodeling in children.

In addition, the importance of histological diagnosis, supported by clinical and radiological diagnoses, must be emphasized, as it contributes to determining the correct treatment plan.

**Figure 13:**
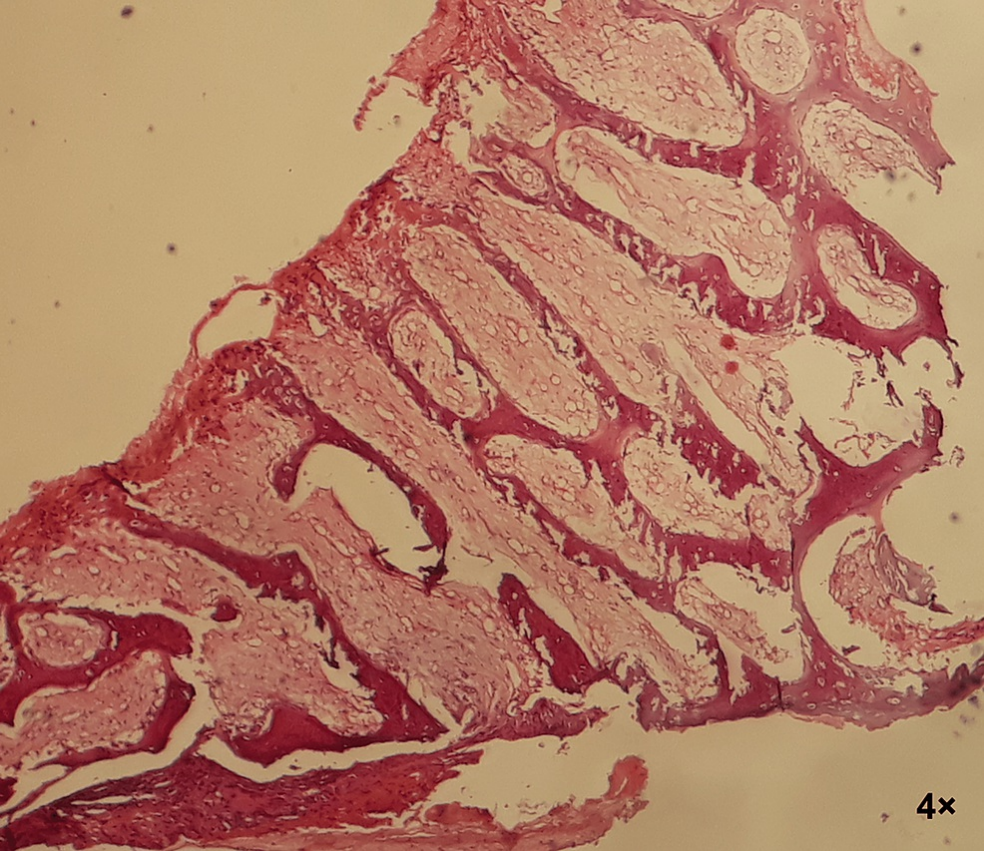
Bone segment from the biopsy misdiagnosed as fibrous dysplasia This pathological diagnosis was not convincing for the medical staff because it is far from clinical findings.

In this case, two biopsies were sent to different laboratories. The first was sent to an external laboratory but the result of the first biopsy was completely different from the differential diagnosis of the medical team, in that it was claimed to be fibrous dysplasia (Figure 13) because the study did not rely on the medical report accompanying the biopsy. The second was sent to the histological and pathological laboratory in the faculty of dentistry, and it confirmed the primary diagnosis with the support of the rest of the diagnostic tools.

## Conclusions

Cysts are common lesions that are frequently found in our practice in oral and maxillofacial surgery. The features in this case (large volume of the cyst, proximity to and containing important structures, extensive harm to the jaw bone, and the young age of the patient) lead us to choose this approach of treatment (marsupialization). Finally, referring to previous literature led to this treatment result.
